# Richness and composition of anuran assemblages from an Amazonian savanna

**DOI:** 10.3897/zookeys.843.33365

**Published:** 2019-05-09

**Authors:** Carlos Eduardo Costa-Campos, Eliza Maria Xavier Freire

**Affiliations:** 1 Universidade Federal do Amapá, Departamento de Ciências Biológicas e da Saúde, Laboratório de Herpetologia, Rodovia Juscelino Kubitschek, km 02, Jardim Marco Zero, CEP 68.903-419, Macapá, AP, Brasil; 2 Programa de Pós-Graduação em Psicobiologia, Universidade Federal do Rio Grande do Norte, Centro de Biociências, Lagoa Nova, CEP 59072-970, Natal, RN, Brasil; 3 Universidade Federal do Rio Grande do Norte, Departamento de Botânica, Ecologia e Zoologia, Campus Central, Laboratório de Herpetologia, Lagoa Nova, CEP 59072-970, Natal, RN, Brasil

**Keywords:** Amapá, eastern Amazon, frog assemblages, tropical forest, amphibians

## Abstract

The Amazonian savannas occupy approximately 150,000 km^2^ of the Brazilian Amazon, occurring in scattered isolated patches over large areas of forest in the states of Amapá, Amazonas, Pará, Roraima and Rondônia. Despite having considerable variation in the Anuran composition between locations and between the savanna’s physiognomies, a systematic and geographically wide sampling has not been performed for the savanna from Amapá yet, located in the north of Brazil, eastern Amazonia. In this perspective, a study was conducted on the richness, composition, diversity, and abundance of Anuran species in a ​​savanna area in Amapá State. For Anuran sampling, we performed 24 samples in four physiognomies (grassland savanna, scrub grassland savanna, parkland savanna, open woodland savanna) through an active and auditory search more than 20 sampling plots of 100 × 50 meters in each physiognomy. Twenty-one (21) species of frogs belonging to five families were registered: Bufonidae, Hylidae, Leptodactylidae, Microhylidae and Phyllomedusidae. Scrub grassland savanna registered a greater number of individuals regarding the species richness by physiognomy. The species rarefaction curve for the total area reached an asymptote, suggesting that the data collection effort was enough to adequately sample the species richness of the area. The Kruskal-Wallis variance analysis revealed significant differences in the species richness and diversity among the physiognomies. The Bray-Curtis similarity analysis grouped the physiognomies into three main groups: open woodland savanna, grassland savanna and scrub grassland savanna and parkland savanna. Through ordering by non-metric multidimensional scaling, the species composition from the savanna anuran assemblage resulted in a separation among three sampled physiognomies with significant differences, indicating differences in assemblage composition of the three sampled physiognomies. The local richness (21 species) corresponds to 14% of the 15 typical species that have strongly associated distribution with the Cerrado from Central Brazil, and 35.6% of 59 typical species of neighboring domains which only marginally occur in the Cerrado, representing a considerable part of frog species richness recorded in the savanna in the eastern portion of the Brazilian Amazon.

## Introduction

The Amazonian savannas are usually flat areas covered by open vegetation composed of herbaceous strata dominated by grasses, with trees and shrubs scattered in different coverage densities (Solbrig 1996). They occupy approximately 267,164 km^2^ with almost 90% of the total area occurs in Bolivia and Brazil, occurring in isolated spots spread over large areas of forest in the states of Amapá, Amazonas, Pará, Roraima and Rondônia (Pires and Prance 1985; Sanaiotti et al. 1997) with smaller areas in Venezuela, Guyana and Suriname (Carvalho and Mustin 2017). The largest continuous savanna block are the Beni savannas in Bolivia (127,096 km^2^); the Guyanan savanna in Brazil, Venezuela and Guyana; the Sipaliwini-Parú savanna in Brazil and Suriname, and the savanna of Amapá in Brazil (13,027 km^2^) (Pennington et al. 2006, Barbosa et al. 2007, Carvalho and Mustin 2017).

The complexity and heterogeneity found in different savanna phytophysiognomies (Eiten 1972, Coutinho 1978), as well as the influence of neighboring domains, reveal the existence of geographic distribution patterns, diversity, richness and abundance of inhabiting species in these areas (Mesquita et al. 2006). With regard to fauna studies and given the great diversity of anuran amphibians from savannas, the number of studies about assemblage ecology in Amazonian savannas has been increasing over the years, addressing different aspects such as the mechanisms that act on the selection and use of habitats and the factors responsible for maintaining diversity, and in the proper assemblage structures (Menin et al. 2007; Menin et al. 2011; Rojas-Ahumada et al. 2012; Landeiro et al. 2014; Dias-Terceiro et al. 2015; Jorge et al. 2016; Ferrão et al. 2018; Ferreira et al. 2018).

In this context, despite the territorial extension and heterogeneity, the Amazon savannas are extremely unfamiliar areas in terms of their frog communities and are highly threatened by the expansion of human activity, therefore it is urgent to conduct inventories on anuran species (Neckel-Oliveira et al. 2000, Barreto et al. 2007, Bertoluci et al. 2007, Brasileiro et al. 2008, Giaretta et al. 2008, Pinheiro et al. 2012, Lima et al. 2017). The strong anthropic pressure, the high degree of endemism and declining savanna frog populations are other factors that justify the importance of inventories (Stuart et al. 2004, Eterovick et al. 2005).

Despite the considerable variation in the anuran composition between locations and between the Cerrado physiognomies (Neckel-Oliveira et al. 2000, Valdujo et al. 2012), a systematic and geographically wide study has not been performed for the Eastern Amazonia savanna. Therefore, an inventory on the anurans in the savanna area of Amapá was performed in order to know the composition, richness, and diversity along the different physiognomies. In this study, we investigated the following questions: (1) what is the species composition of anurans in the study area? (2) Do the species richness, diversity, and equitability differ between different types of savanna physiognomies? Additionally, we compared our findings with studies conducted in other Amazon savannas and well-sampled areas in the Cerrado in central Brazil.

## Materials and methods

### Study area

**Figure 1.**
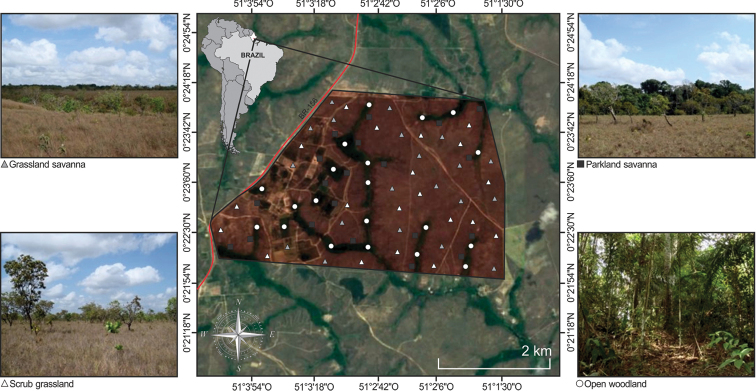
Maps showing the Amapá state and sampling sites in the Experimental Field of the Brazilian Agricultural Research Corporation – EMBRAPA, municipality of Macapá, Amapá State, northern Brazil.

The Amapá savanna occupies a narrow longitudinal band of approximately 140,000 km^2^, which corresponds to 7.2% of the total area of the Amapá State territory (Mustin et al. 2017), is characterised by a mosaic of areas with open woody vegetation, areas with a denser woody shrub layer, and open grassy areas, and by seasonally flooded areas in the transition zone with floodplains (Costa-Neto et al. 2017). In this area, specifically in the Experimental Field of the Brazilian Agricultural Research Corporation – EMBRAPA (00°23'5"N; 51°02'2"W), the present study was conducted in a total area of 1,381 hectares and a perimeter of 15,080 m (Figure 1).

Four savanna physiognomies were sampled (cf. Miranda et al. 2003; Melém-Júnior et al. 2003): (1) grassland savanna (GS), characterized by extensive grass fields, abundant herbaceous stratus and woody stratus well dispersed by the continuous herbaceous stratus and sparse dwarf trees (< 1.0 m high); (2) scrub grassland savanna (SG), with herbaceous stratus covered by grass with a large density and tree stratus with irregular presence and sparse small trees (< 3.8 m high); (3) parkland savanna (PS), with less developed herbaceous stratus and thicker arboreal tree cover (< 4.3 m high) forming a discontinuous canopy; and (4) open woodland savanna (OW), with and absent of herbaceous stratus with higher density, forming a closed canopy (> 2.0 m high).

**Figure 2.**
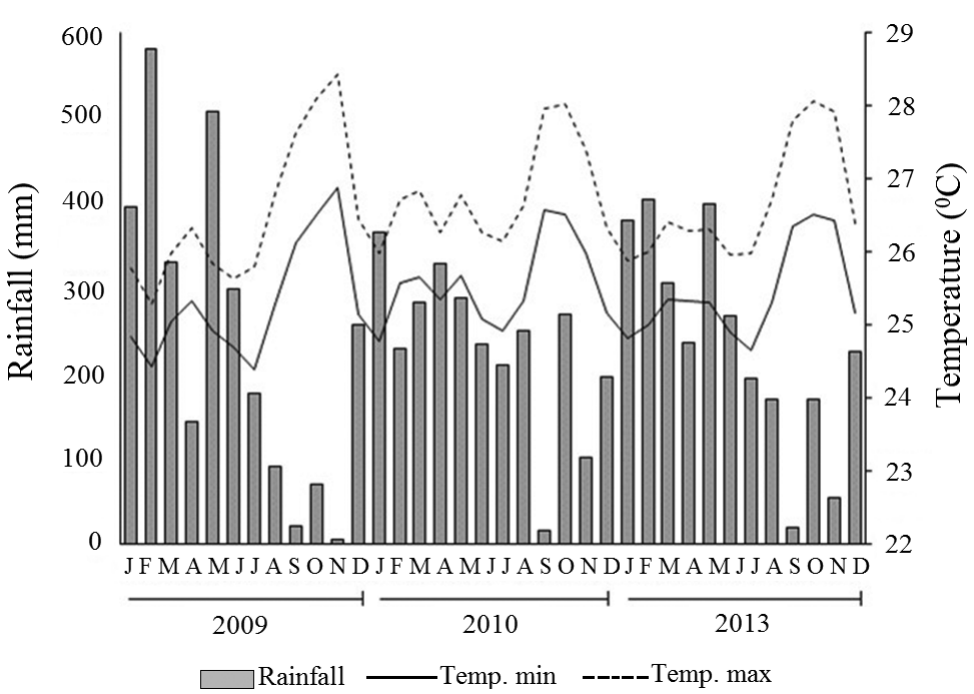
Rainfall data and minimum and maximum temperatures at the Experimental Field of the Brazilian Agricultural Research Corporation – EMBRAPA, municipality of Macapá, Amapá State, northern Brazil between January and December 2009, 2010, and 2013.

The predominant climate type is Ami (tropical rainy climate with short dry period) according Köppen-Geiger classification. Due to the concentration of rainfall in six consecutive months (January – July), the climate throughout the year can be typically recognized in only two seasons: a quite clear dry season and a rainy season with high rainfall. Regarding the monthly average temperature, the minimum is 24.4 °C and maximum 28.4 °C (Alvares et al. 2013). Summarized data is displayed in Figure 2.

The terrain is flat or gently hilly on soils that occur in two main forms, being oxisols and quartz sands. The savanna domain occurs on two basic types of terrain: crystalline or sedimentary plateaus and interplateau depressions. In general, the regions of plateaus predominate in the interfluvia wider savanna forms, while the interplateau depressions occur in denser cerrado (Aguiar et al. 2004).

### Sampling design

For the anuran sampling plot 24 incursions into each physiognomy were conducted, 12 in the rainy season and 12 in the dry season. Twenty (20) sampling units were established in each physiognomy (grassland savanna, scrub grassland savanna, parkland savanna and open woodland savanna), arranged according to the availability of water bodies. Each sample unit was represented by a portion of 100×50 meters (0.5 ha), at least 500 meters away from each other. Active and auditory visual searches were conducted in these sampling units (Crump and Scott 1994, Heyer et al. 1994, Zimmerman 1994). The sampling effort was the same in the four physiognomies to enable comparisons. The species taxonomy applied follows the Brazilian Society of Herpetology (SBH), according to Segalla et al. (2016) and Dubois (2017).

The active visual and auditory search was performed in each sampling plot by four researchers from 18:00 to 00:00. The number of individuals of each species calling activity was recorded every 10 minutes. The sampling effort was calculated by multiplying the number of hours in the field by the number of researchers involved in the collection in both methods, resulting in a sampling effort of 1,920 hours/man. All collected specimens were anesthetized and killed, fixed in 10% formaldehyde and stored in 70% ethanol. Voucher specimens are deposited at the Herpetological Collection of the Universidade Federal do Amapá (UNIFAP), under the care of Carlos Eduardo Costa Campos (CECCAMPOS) (Appendix 1).

### Data analysis

The amphibian species diversity of between the four studied savanna physiognomies was calculated through the Shannon-Wiener diversity Index (Krebs 1999), where *H*’ = species diversity index, *pi = Ni / N* = probability that an individual belongs to species *i* in total “*S*” species, *Ni* = total number of individuals of the species *i*, *N* = total number of species.

The equitability index was calculated by the ratio between the diversity obtained and the maximum diversity, where *j* = equitability, *H*’ = achieved diversity. *Hmax*’ = maximum diversity. This index shows the population homogeneity or how the species are represented by the number of individuals of each species in the assemblage (Magurran 2004). This index ranges from *0*, when a species excels in abundance, to *1*, when species are equally abundant in the environment.

To analyze the anuran species richness, accumulation curves of species were built based on the number of individuals and number of samples (Gotelli and Colwell 2001, 2011) using the EstimateS 9.1 program (Colwell 2013) with 1,000 randomizations. Considering the diverse richness estimators available, we chose to use the Jackknife algorithm first order based on its performance when compared to other estimators (Magurran 2004; Hortal et al. 2006; Reese et al. 2014)

The possible differences in the richness variations, diversity, and equitability in the different physiognomies were verified through one-way ANOVA, Kruskal-Wallis and Dunn tests a posteriori (Zar 2010). All the analyses were performed through the BioEstat 5.0 (Ayres et al. 2007) and PAST 2.09 (Hammer et al. 2001) programs. The values were considered significant at p < 0.05.

To verify if the assemblage composition differs between physiognomies, a cluster analysis (Cluster Analysis) and spatial Non-Metric Multidimensional Scaling (NMDS) were performed. The differences between the species composition and physiognomies were evaluated using an Analysis of Similarity ANOSIM (Clarke 1993). A percentage break analysis of the Similarity (SIMPER) considering the Bray-Curtis similarity index was used to detect which frog species are responsible for the differences between the groups.

## Results

### Species composition

**Figure 3.**
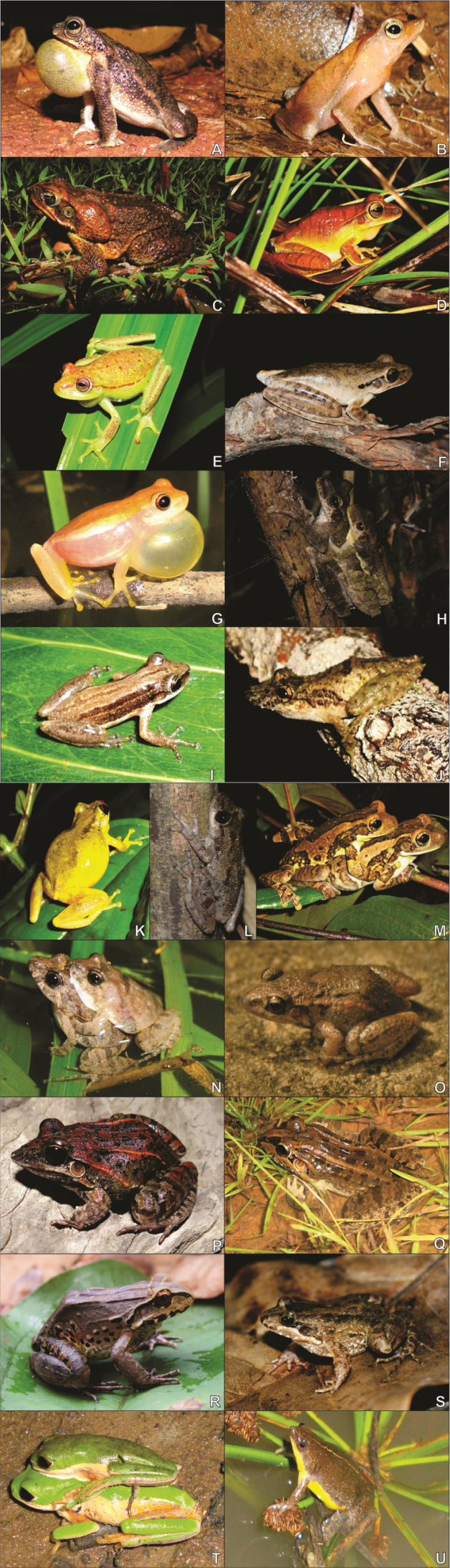
Anurans recorded in the savanna area from the Experimental Field of the Brazilian Agricultural Research Corporation – EMBRAPA, municipality of Macapá, Amapá State, northern Brazil: **A***Rhinellamajor***B***Rhinella* sp. **C***R.marina***D***Boanamultifasciata***E***B.punctata***F***B.raniceps***G**Dendropsophuscf.walfordi**H***Osteocephalustaurinus***I***Scinaxfuscomarginatus***J***S.nebulosus***K***S.ruber***L***S.x-signatus***M***Trachycephalustyphonius***N***Pseudopaludicolaboliviana***O***Adenomerahylaedactyla***P***Leptodactylusfuscus***Q***L.macrosternum***R***L.pentadactylus***S***L.podicipinus***T***Pithecopushypochondrialis*, and **U***Elachistocleishelianneae*.

We recorded 21 anuran species belonging to the following families were obtained: Bufonidae (3 species), Hylidae (10 species), Leptodactylidae (6 species); Microhylidae (1 species), and Phyllomedusidae (1 species) (Table 1, Figure 3). From the recorded species in the study area, three representatives of the Hylidae, Leptodactylidae and Microhylidae families (*Scinaxfuscomarginatus, Pseudopaludicolaboliviana*, *Elachistocleishelianneae*, respectively) were recently reported for the first time in the Amapa state by these three studies (Costa-Campos and Freire 2014, Costa-Campos and Freire 2015, Costa-Campos et al. 2016).

**Table 1.** List of anuran species registered at the savanna area in Amapá state, municipality of Macapá. Gray blocking denotes anurans sampled in each physiognomies. Sampled physiognomies: **GS** grassland savanna; **SG** scrub grassland savanna; **PS** parkland savanna; **OW** open woodland savanna. **N** number of individuals recorded. Number of species per family in parentheses.

Family/Species	Sampled physiognomies				N
	GS	SG	PS	OW	
**Bufonidae (3)**					
*Rhinellamajor* (Muller & Helmich, 1936)					**10**
*Rhinellamarina* (Linnaeus, 1758)					**5**
*Rhinella* sp.					**14**
**Hylidae (10)**					
*Boanamultifasciata* (Günther, 1859)					**20**
*Boanapunctata* (Schneider, 1799)					**18**
*Boanaraniceps* Cope, 1862					**15**
Dendropsophuscf.walfordi (Bokermann, 1962)					**8**
*Osteocephalustaurinus* Steindachner, 1862					**10**
*Scinaxfuscomarginatus* (A. Lutz, 1925)					**1**
*Scinaxnebulosus* (Spix, 1824)					**32**
*Scinaxruber* (Laurenti, 1768)					**18**
*Scinaxx-signatus* (Spix, 1824)					**12**
*Trachycephalustyphonius* (Linnaeus, 1758)					**27**
**Leptodactylidae (Leiuperinae) (1)**					
*Pseudopaludicolaboliviana* Parker, 1927					**14**
**Leptodactylidae (Leptodactylinae) (5)**					
*Adenomerahylaedactyla* (Cope, 1868)					**32**
*Leptodactylusfuscus* (Schneider, 1799)					**30**
*Leptodactylusmacrosternum* Miranda-Ribeiro, 1926					**5**
*Leptodactyluspentadactylus* (Laurenti, 1768)					**8**
*Leptodactyluspodicipinus* (Cope, 1862)					**12**
**Microhylidae (Gastrophryninae) (1)**					
*Elachistocleishelianneae* Caramaschi, 2010					**14**
**Phyllomedusidae (1)**					
*Pithecopushypochondrialis* (Daudin, 1800)					**17**

### Sample efficiency: species rarefaction curve and richness estimators

**Figure 4.**
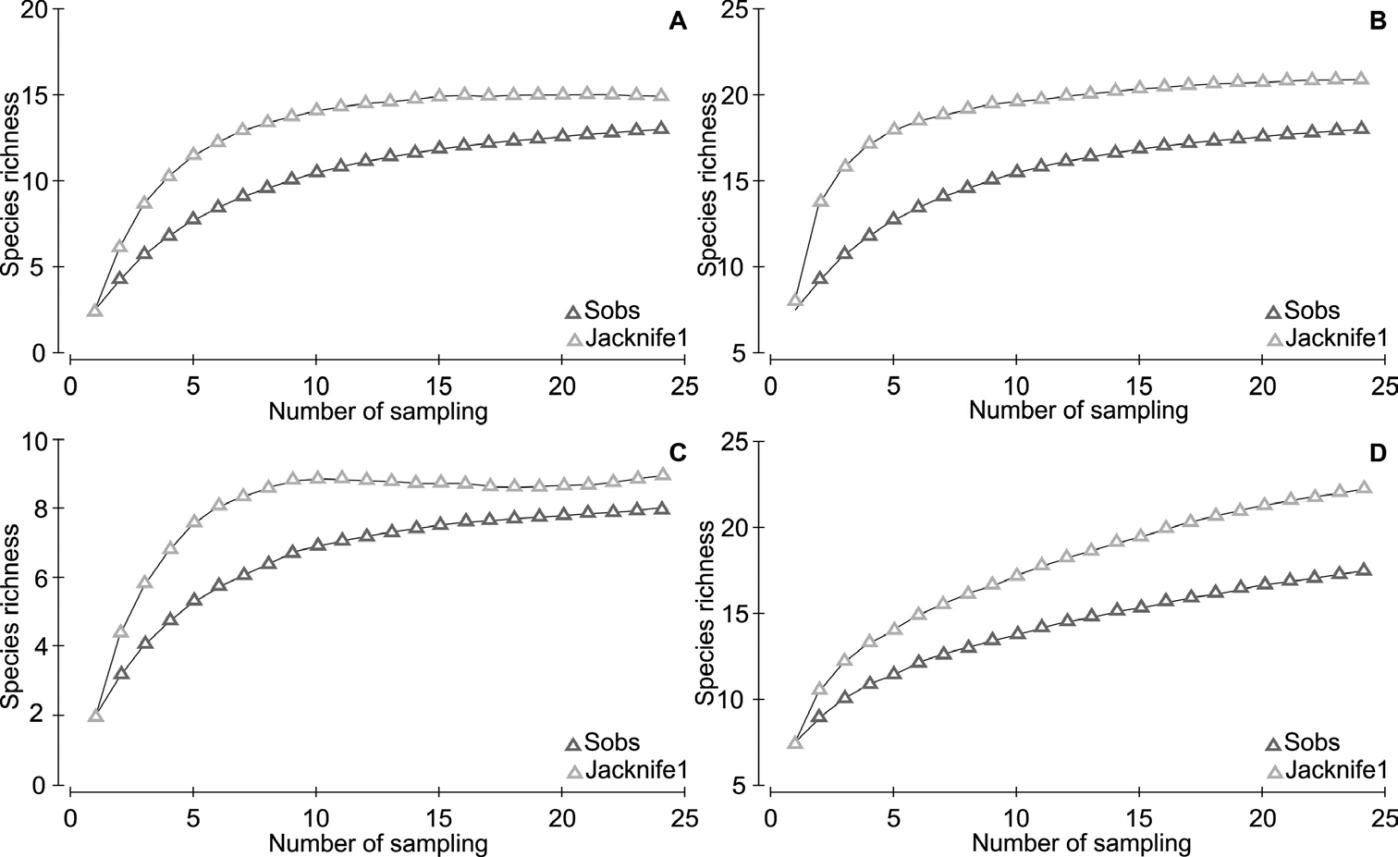
The species accumulation curves sampled in the savanna area from the Experimental Field of the Brazilian Agricultural Research Corporation – EMBRAPA, municipality of Macapá, Amapá State, northern Brazil, for each of the sampled physiognomies: **A** grassland savanna **B** scrub grassland savanna **C** parkland savanna **D** open woodland savanna, based on the number of individuals representing the observed (Sobs) and estimated species richness (Jackknife 1).

A slight tendency toward stabilization can be observed in the species accumulation curve (Figure 4), suggesting that the data collection effort was enough to adequately sample the species richness of the studied area. The estimated richness for the study area provided by the richness estimators was 21 species.

### Species diversity and equitability

The species diversity obtained the sampled physiognomies ranged from *H* ‘= 0.814 to *H*’ = 2.728, and the equitability ranged from 0.587 to 1.887. The highest diversity of species was recorded at scrub grassland savanna (*H* ‘= 2.459; N = 19 species), followed by grassland savanna (*H* ‘= 2.276; N = 13 species). The Kruskal-Wallis variance analysis revealed significant differences between the species richness, diversity, dominance (1-D) and equitability between the sampled physiognomies (p < 0.0001). The Dunn test results performed later showed significant differences in species richness, diversity and equitability between the Amapá savanna physiognomies (Table 2).

**Table 2.** Kruskal-Wallis variance analysis and the Dunn test performed later between the species richness, diversity and equitability between the savanna physiognomies in Amapá. The significant results are in bold (p < 0.05).

Physiognomies	Richness	H’	J
Grassland savanna and scrub grassland savanna	***4.318***	***4.044***	*2.173*
Grassland savanna and parkland savanna	*0.093*	*0.331*	*0.150*
Grassland savanna and open woodland savanna	*1.479*	*1.619*	*1.795*
Scrub grassland savanna and parkland savanna	***4.412***	***4.375***	*2.323*
Scrub grassland savanna and open woodland savanna	***5.798***	***5.663***	***3.969***
Parkland savanna and open woodland savanna	*1.386*	*1.287*	*1.645*

### Similarity and non-metric Multidimensional Scaling (MDS) between different physiognomies from the studied savanna

**Figure 5.**
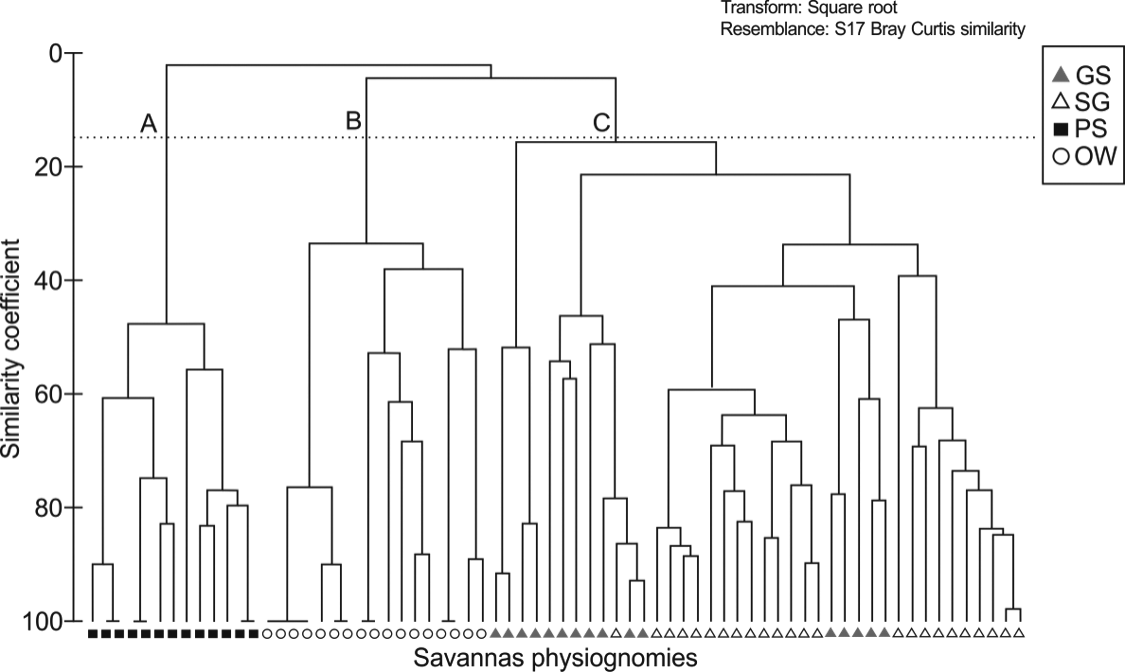
Dendrogram of similarity between the savanna area physiognomies, sampled in the Experimental Field of the Brazilian Agricultural Research Corporation – EMBRAPA, municipality of Macapá, Amapá State. Group A (open woodland savanna, OW), Group B (Parkland savanna, PS), and Group C (grassland savanna, GS and scrub grassland savanna, SG).

The similarity analysis from the Bray-Curtis index separated the physiognomies into three main groups: (A) open woodland savanna, (B) grassland savanna and scrub grassland savanna (C) parkland savanna. The group (A) is characterized by the higher *Rhinella* sp. occurrence frequency. For the group (B), the most frequent species were *P.hypochondrialis* and *L.fuscus*. In addition, the last group (C) is characterized by the high frequency of occurrence of *B.punctata* (Figure 5).

The similarity analysis (SIMPER) evaluated the contribution of each species and showed that the average similarity within physiognomies was 27.48 for the grassland savanna, 48.84 for scrub grassland savanna, 58.70 open woodland savanna and 47.02 for parkland savanna. For the grassland savanna, the *L.fuscus* species contributed the most to the average similarity with 53.64, followed by *B.multifasciata* (13.94) and *T.typhonius* (13.64). For scrub grassland savanna, the *P.hypochondrialis* species contributed the most to the average similarity with 26.68 of contribution, followed by *L.fuscus* (18.43) and *S.nebulosus* (16.25). For the open woodland savanna, the *Rhinella* sp. (88.40) and *A.hylaedactyla* species (11.27) were those that contributed the most to the average similarity. For the parkland savanna, *B.punctata* (81.84) and *B.raniceps* species (8.44) contributed the most to the average similarity.

The assemblage average dissimilarity between physiognomies was larger between: open woodland savanna and parkland savanna (99.53); the scrub grassland savanna and the open woodland savanna (97.48); the grassland savanna and the open woodland savanna (96.45); the grassland savanna and the parkland savanna (96.22); the scrub grassland savanna and the parkland savanna (94.85); and lower between grassland savanna and scrub grassland savanna (75.18). Regarding the total differences, the species that contributed most to the average dissimilarity between areas were: *Rhinella* sp. (open woodland savanna and parkland savanna, 32.0%); *B.punctata* (grassland savanna and parkland savanna, 21.7%); and *P.hypochondrialis* (grassland savanna and scrub grassland savanna, 17.37%).

**Figure 6.**
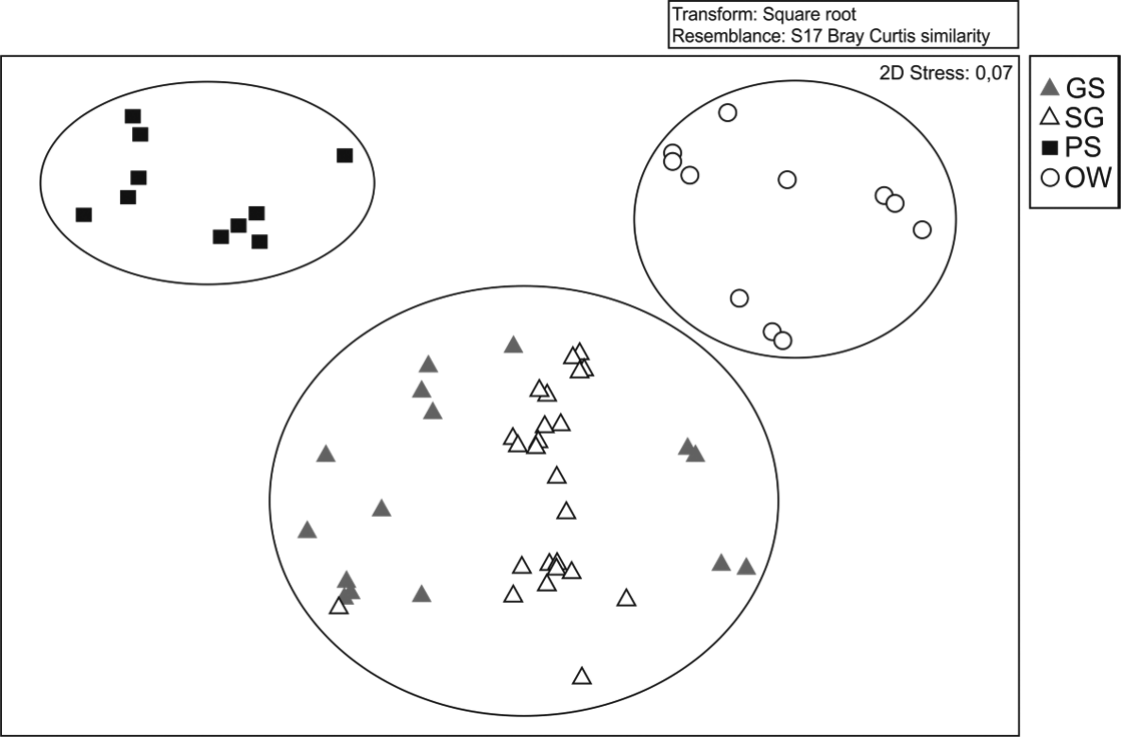
Order by MDS of physiognomies in the savanna area physiognomies, sampled in the Experimental Field of the Brazilian Agricultural Research Corporation – EMBRAPA, municipality of Macapá, Amapá State generated from the Bray-Curtis similarity matrix. Grassland savanna (GS); scrub grassland savanna (SG); parkland savanna (PS); open woodland savanna (OW).

Through the MDS constructed from the Bray-Curtis similarity matrix, it has been shown that the anuran assemblage in the study area is distributed between three sampled physiognomies, formed by 1) grassland savanna and scrub grassland savanna, 2) parkland savanna, and 3) open woodland savanna, with significant differences (ANOSIM, R = 0.823, p <0.001) and stress level of 0.07. In this way, the assemblage formed distinct groups in the MDS, indicating differences in the assemblage species composition of the four physiognomies (Figure 6).

## Discussion

The registered richness supports the estimated richness of 18-43 species in well sampled locations in the Cerrado of Central Brazil (Brasileiro et al. 2005, Vasconcelos and Rossa-Feres 2005, Santos et al. 2007, Brasileiro et al. 2008, Ribeiro-Jr and Bertoluci 2009, Araújo and Almeida-Santos 2011) and in Amazonian savannas (Neckel-Oliveira et al. 2000, Pinheiro et al. 2012, Bitar et al. 2012, Neckel-Oliveira et al. 2012, Lima et al. 2017). The local richness corresponds to 14% of the 150 species which have distribution strongly associated with the Cerrado, and 35.6% of the 59 typical species of neighboring domains and which only marginally occur in the Cerrado (Valdujo et al. 2011, 2012), representing 21 anuran species recorded in the savannas of the eastern portion of the Brazilian Amazon.

The anuran species richness of the studied savanna varied in different physiognomies, where the open formations (e.g., grassland savanna) had greater richness, followed by forest formations (e.g., parkland and open woodland savanna). This is probably a reflex of the proportion of abundant and rare species in each physiognomy (Magurran 2004). These data agree with other studies, which suggest that the open formations tend to have higher richness when compared to forest formations of the Domain Cerrado (Maffei et al. 2011). Studies carried out by Strüssmann et al. (2000), Brandão and Araújo (2002) and Valdujo et al. (2011) found higher anuran richness in open savanna formations than in the gallery forest. This is due to the fact that the reproduction of most savanna species occurs in open areas, and as the availability of breeding sites is one of the most important factors in the selection of habitat for anurans, higher species richness and abundance of individuals is expected in open physiognomies (Colli et al. 2002, Bastazini et al. 2007).

On the other hand, in the study performed by Neckel-Oliveira et al. (2000) in an area of the Amazonian savanna in the municipality of Santarém, by Pinheiro et al. (2012) in the Carajás region, both in the state of Pará, and by Lima et al. (2017) in the Amazonian Savanna of the Rio Curiaú Environmental Protection Area in Amapá, higher richness and abundance of anuran species were found in forested environments. Several studies report that species of open environments can use forested areas as sites for foraging, migration, reproduction, or refuge (Brassaloti et al. 2010, Oda et al. 2016, Santos and Conte 2016).

Furthermore, several studies have shown that complex and heterogeneous environments facilitate the coexistence of more species when compared to homogeneous environments (Conte and Rossa-Feres 2007, Santos et al. 2007, Vasconcelos et al. 2009, Silva et al. 2011). In addition to the heterogeneity and biodiverse of the Cerrado (Silva et al. 2006), differences in their composition and species richness between various sampled locations must have been favored by the contact with four major phytogeographic domains from South America: Amazonia, Atlantic Forest, Caatinga, and Chaco (Joly et al. 1999).

Despite the species cumulative curve having a tendency to stabilize, the possibility of local richness expansion is not unlikely, but as the study continues, an increased effort would contribute very slowly to adding to the species richness, as evidenced by richness estimators. This highlights the importance of accomplishing inventories with the association of different sampling methods for a more complete knowledge of anurans (Greenberg et al. 1994, Brown 1997, Maritz et al. 2007; Ribeiro-Jr et al. 2008), as the species richness is closely related to the sampling effort.

According to Santos (2006), accumulation curves are excellent for evaluating the inventories efficiency in the record of all species of certain sites or habitats. Similar to the rarefaction for the total area, the species accumulation curves for all physiognomies also stabilized; however, for the tree savanna stabilized in a relatively low species richness, this indicates that the additional sample effort would be necessary in the forest environments. In this context, three non-mutually exclusive factors and associated to the canopy cover structure may be responsible for the different richness patterns and frog species composition observed in the studied physiognomies: 1) some species ability in colonizing environments characterized by sparser vegetation; 2) the species distinct physiological tolerances concerning water temperature, light intensity and humidity near the soil surface; and 3) specific microenvironment dependence on specific sites for breeding.

In terms of diversity by physiognomies, the greatest diversity was found in scrub grassland savanna. The higher diversity recorded in the scrub grassland savanna corroborates comparative studies of the species diversity of open formations with grasslands backgrounds, savanna and forest in the Cerrado (Brasileiro et al. 2005, Araújo et al. 2009, Araújo and Almeida-Santos 2011, Valdujo et al. 2011). The diversity and equitability rates showed significant differences between sampled physiognomies. This result was expected due to the differences present in the physiognomies in the environmental heterogeneity (Vasconcelos et al. 2009, 2010), as this heterogeneity has an important role in determining species richness and in the assemblage structure by providing environments and several conditions for species with different ecophysiological requirements (Huston 1994, Buckley and Jetz 2007).

The obtained Bray-Curtis index seems coherent, presenting groupings that reflect differences between the main sampled physiognomies and revealing greater similarity between the grassland savanna and the scrub grassland savanna. Both physiognomies differ in the floristic composition of the dominant tree species, shrub and herbaceous substrate, and topography (Batalha 2011), and these differences have probably influenced the obtained results. However, geographically close areas generally exhibit similar structural features, which support similar assemblages in their richness and diversity (Dixo and Verdade 2006). Even though they do not favor species with more limited needs, the parkland savanna and open woodland savanna physiognomies, with lower structural complexity, are relatively favorable for structuring frog assemblages, thus potentially contributing to the preservation of regional diversity in mosaic vegetation areas (Gardner et al. 2006).

The Amapá savanna although scarcely known, may suffer from the agricultural expansion of grain production, extensive cattle ranching and urban growth, leading to habitat loss and vegetation fragmentation. Coupled with the wide diversity of anurans found in the area and the finding of new species and new records for Amapá State make Amapá savanna a hotpoint for anurans within the Amazon Forest hotspot (Silva and Bates 2002; Lima et al. 2017, Mustin et al. 2017) and, consequently, a place for the implementation of priority conservation measures aiming the increase of the protected area.

## Appendix I

**Voucher specimens**

Voucher specimens are deposited at the Herpetological Collection of the Universidade Federal do Amapá (UNIFAP), under the care of Carlos Eduardo Costa Campos (CECCAMPOS).

BUFONIDAE

*Rhinellamajor* – CECCAMPOS 0747.

*Rhinella* sp. – CECCAMPOS 0799-0800, 0805-0806.

*Rhinelamarina* – CECCAMPOS 0668.

HYLIDAE

*Boanamultifasciata* – CECCAMPOS 0035, 0132, 0545.

*Boanapunctate* – CECCAMPOS 0050, 0130, 0550, 0554, 0600.

*Boanaraniceps* – CECCAMPOS 0486.

Dendropsophuscf.walfordi – CECCAMPOS 0765-0769.

*Osteocephalustaurinus* – CECCAMPOS 0676.

*Scinaxfuscomarginatus* – CECCAMPOS 0037.

*Scinaxnebulosus* – CECCAMPOS 0575-0580.

*Scinaxruber* – CECCAMPOS 0745, 0747, 0761.

*Scinaxx-signatus* – CECCAMPOS 0321, 0683-0684.

*Trachycephalustyphonius* – CECCAMPOS 0739-0740.

LEPTODACTYLIDAE

*Pseudopaludicolaboliviana* – CECCAMPOS 1153.

*Adenomerahylaedactyla* – CECCAMPOS 0047, 0548, 0560, 0609.

*Leptodactylusfuscus* – CECCAMPOS 0586, 0589, 0748, 0749, 0762.

*Leptodactylusmacrosternum* – CECCAMPOS 0485, 0582.

*Leptodactyluspentadactylus* – CECCAMPOS 0670, 0737.

*Leptodactyluspodicipinus* – CECCAMPOS 0048, 0049, 0606, 0753, 0772.

MICROHYLIDAE

*Elachistocleishelianneae* – CECCAMPOS 0122, 0227.

PHYLLOMEDUSIDAE

*Pithecopushypochondrialis* – CECCAMPOS 0546, 0572, 0733.
